# Successful Awake Blind Nasal Intubation for Airway Management of a Giant Parapharyngeal Space Tumor in a Resource‐Limited Setting

**DOI:** 10.1155/cria/7618732

**Published:** 2026-10-05

**Authors:** Markos Muka, Aschalew Besha, Kanbiro Gedeno

**Affiliations:** ^1^ Department of Anesthesia College of Medicine and Health Sciences Arba Minch University Arba Minch Ethiopia amu.edu.et; ^2^ Department of Anesthesia College of Medicine and Health Sciences Hawassa University Hawassa Ethiopia hu.edu.et

**Keywords:** awake blind nasal intubation, awake tracheal intubation, case report, difficult airway, parapharyngeal space tumor, resource-limited setting

## Abstract

**Introduction:**

Parapharyngeal space (PPS) tumors are rare and account for approximately 0.5% of all head and neck tumors. Their close proximity to the upper airway can result in significant anatomical distortions, making airway management challenging. Awake tracheal intubation is recommended when a difficult airway is anticipated; however, advanced airway devices such as flexible fiberoptic bronchoscopes and videolaryngoscopes may not be available in low‐resource settings. Awake blind nasal intubation (ABNI), although rarely practiced today, may be a feasible alternative in carefully selected patients.

**Objective:**

We report successful airway management with ABNI in a patient with PPS tumor in a resource‐limited setting.

**Case Presentation:**

A 45‐year‐old man presented with a progressively enlarging giant PPS tumor, associated with dysphagia, snoring, and oral airway obstruction. Preoperative airway evaluation revealed Mallampati Class III, preserved mouth opening, and unrestricted cervical spine mobility; however, the large intraoral mass suggested a potentially difficult airway. Because fiberoptic bronchoscopy and videolaryngoscopy were unavailable, ABNI was planned while maintaining spontaneous ventilation, and emergency surgical tracheostomy was performed as a rescue strategy. Airway topicalization was achieved using 2% lidocaine gargle and intravenous lidocaine, together with nasal vasoconstriction using adrenaline‐soaked gauze. A lubricated 6.0‐mm cuffed endotracheal tube was successfully inserted through the left nostril on the first attempt, without hypoxemia, airway trauma, or hemodynamic instability. General anesthesia was administered uneventfully, and complete tumor excision was successfully performed. The patient recovered without perioperative complications and was discharged on postoperative day six.

**Conclusion:**

This case demonstrates that ABNI may represent a practical alternative for selected patients with anticipated difficult airways due to PPS tumors when advanced airway equipment is unavailable. Careful patient selection, meticulous airway assessment, adequate topical anesthesia, maintenance of spontaneous ventilation, and preparation for emergency surgical airway management remain essential. Although this case illustrates technical feasibility, broader conclusions regarding safety or effectiveness cannot be drawn from a single case.

## 1. Introduction

Parapharyngeal space (PPS) tumors are rare neoplasms that account for approximately 0.5% of all head and neck tumors. Owing to the complex anatomy of the PPS, which contains major blood vessels, cranial nerves, salivary tissue, and lymphatic structures, these tumors present significant diagnostic, surgical, and anesthetic challenges. Approximately 70%–80% of PPS tumors are benign, with pleomorphic adenomas of the deep lobe of the parotid gland, schwannomas, and paragangliomas being the most frequently encountered histological subtypes. Their deep anatomical location often delays diagnosis until they become sufficiently large to produce symptoms related to compression of adjacent structures [1–4].

As PPS tumors enlarge, they may displace or compress the oropharynx, hypopharynx, larynx, or upper airway, resulting in dysphagia, snoring, voice changes, obstructive sleep apnea, and progressive airway obstruction. Consequently, airway management during anesthesia may be particularly challenging because induction of general anesthesia can precipitate complete airway collapse, creating a potentially catastrophic “cannot intubate, cannot oxygenate” (CICO) situation. Therefore, comprehensive preoperative airway assessment and meticulous planning are essential for minimizing perioperative risks [3, 5].

Current difficult airway guidelines from the American Society of Anesthesiologists (ASA) and the Difficult Airway Society (DAS) recommend awake tracheal intubation (ATI) when difficult airway management is anticipated and loss of spontaneous ventilation can have serious consequences [6, 7]. Flexible fiberoptic bronchoscopy remains one of the most commonly used techniques for awake intubation, when expertise and equipment are available. However, videolaryngoscopy has also become an accepted first‐line option for ATI in selected patients, and the choice of technique should depend on patient anatomy, operator experience, and available resources rather than a single “gold standard” approach [6–8].

Advanced airway devices, such as flexible fiberoptic bronchoscopes and videolaryngoscopes, may not be readily available in many low‐ and middle‐income countries, including resource‐limited hospitals. Under these circumstances, anesthesiologists may need to rely on alternative airway management techniques, while ensuring adequate preparation for emergency surgical airway access. Although awake blind nasal intubation (ABNI) has largely been replaced by fiberoptic‐guided techniques in contemporary practice, it may remain a useful rescue or alternative technique in carefully selected patients when advanced airway equipment is unavailable and when clinicians have sufficient experience. Nevertheless, ABNI has recognized limitations, including lower success rates, epistaxis, airway trauma, and the possibility of repeated unsuccessful attempts, emphasizing the importance of careful patient selection and backup airway planning [6–9].

Published reports describing ABNI for giant PPS tumors remain limited, particularly in low‐resource healthcare settings, where access to advanced airway technologies is restricted. Sharing such experiences contributes to the growing body of evidence on context‐appropriate airway management strategies while highlighting the importance of individualized decision‐making based on available resources, patient characteristics, and multidisciplinary planning [3, 5, 9].

Here, we report the successful anesthetic management of a 45‐year‐old man with a giant PPS tumor, who underwent tumor excision under general anesthesia following successful ABNI in a resource‐limited setting. This case illustrates the importance of comprehensive airway assessment, maintenance of spontaneous ventilation during airway management, thorough preparation for rescue surgical airways, and careful multidisciplinary planning when managing anticipated difficult airways in environments with limited access to advanced airway equipment.

## 2. Case Presentation

This case was reported in accordance with SCARE (Surgical CAse REport) guidelines [10]. The completed SCARE checklist is provided in Supporting File (SCARE‐Checklist‐2025.docx).

### 2.1. Patient Information

A 45‐year‐old Ethiopian male (height: 176 cm; weight: 68 kg; body mass index [BMI]: 21.9 kg/m^2^) presented to the ear, nose, and throat (ENT) clinic with a progressively enlarging right‐sided oropharyngeal mass of six months’ duration. The swelling initially appeared as a small painless intraoral lesion that gradually increased in size, causing progressive upper airway obstruction and prompting referral for surgical excision.

The patient complained of progressive dysphagia, predominantly solid foods, persistent snoring during sleep, difficulty breathing through the mouth, and sensation of a foreign body in the throat. He denied experiencing dyspnea at rest, stridor, voice changes, weight loss, fever, or recurrent bleeding from the mass.

The patient’s medical history was unremarkable. He had no history of hypertension, diabetes mellitus, cardiovascular disease, chronic respiratory illness, surgery, anesthesia exposure, medication use, smoking, alcohol consumption, or known drug allergies. Family history was noncontributory.

### 2.2. Clinical Findings

On admission, the patient was comfortable at rest, without respiratory distress, and maintained spontaneous ventilation while breathing room air.

General examination revealed a well‐nourished male with stable vital signs throughout the preoperative period (Table 1).

**TABLE 1:** Preoperative vital signs during hospital admission.

Vitals	At admission	Day before surgery	Preoperative evaluation
Blood pressure	108/68 mmHg	112/67 mmHg	110/70 mmHg
Heart rate	70 bpm	73 bpm	72 bpm
Respiratory rate	16/min	16/min	18/min
Temperature	36.7°C	36.6°C	36.7°C

Oral examination revealed a large parapharyngeal mass that arose predominantly from the right oropharyngeal wall and extended across the midline, occupying a substantial portion of the oropharyngeal cavity. The lesion displaced the soft palate and narrowed the oral airway, significantly limiting the visualization of the posterior pharyngeal structures (Figures 1 and 2). No active bleeding or ulceration of the mass was observed.

**FIGURE 1:**
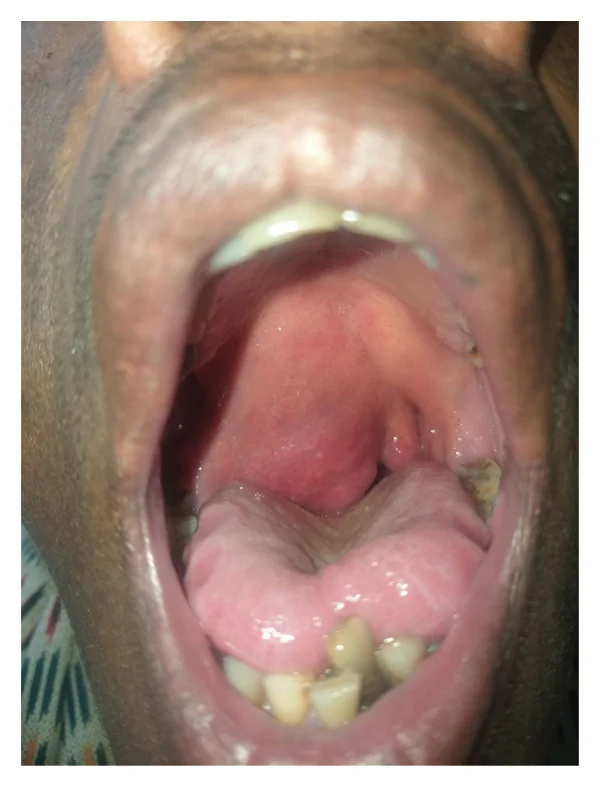
Intraoral view demonstrating a giant right‐sided parapharyngeal space tumor causing marked medial displacement of the oropharyngeal structures and significant narrowing of the upper airway. Preoperative intraoral examination. Abbreviation: PPS, parapharyngeal space. Written informed consent for the publication of clinical details and accompanying images was obtained from the patient.

**FIGURE 2:**
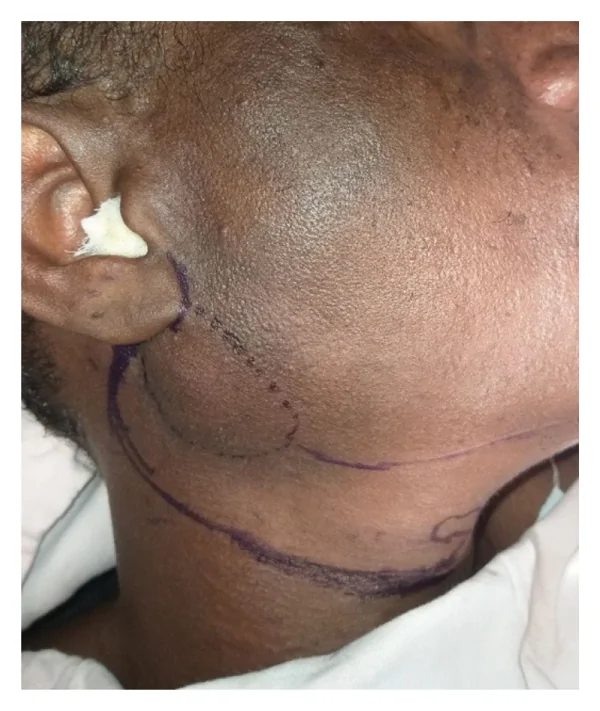
External view of the neck showing a large right‐sided parapharyngeal space mass extending from the mandibular angle to the upper cervical region, producing obvious facial and neck asymmetry. Preoperative external mandibular examination. Abbreviation: PPS, parapharyngeal space. Written informed consent for the publication of clinical details and accompanying images was obtained from the patient.

Neck examination revealed normal cervical spine mobility without limitations of flexion, extension, or lateral rotation. No cervical lymphadenopathy was detected.

### 2.3. Preoperative Airway Assessment

Because a difficult airway was anticipated, a comprehensive airway evaluation was performed.

Airway assessment demonstrated the following: Mallampati Class III, mouth opening greater than three finger breadths, adequate mandibular protrusion, full cervical spine mobility, no limitation of temporomandibular joint movement, no facial deformity, large intraoral parapharyngeal mass causing marked narrowing of the oropharyngeal airway, thyromental distance of 7 cm, and sternal distance of 12.5 cm.

Although conventional bedside airway assessment suggested only moderate difficulty, the extensive intraoral tumor substantially increased the risk of difficult laryngoscopy, difficult tracheal intubation, and potential airway obstruction following the induction of general anesthesia.

### 2.4. Diagnostic Assessment

Routine laboratory investigations were within the acceptable limits for surgery (Table 2). Hemoglobin concentration was 16.6 g/dL, platelet count was 259 × 10^3^/μL, coagulation profile was normal (PT 14.1 s, INR 1.18), and renal function was preserved.

**TABLE 2:** Preoperative laboratory investigations.

Investigation	Result	Reference range
Hemoglobin	16.6 g/dL	12.5–16.3
Hematocrit	48.7%	36.7–47.1
WBC	8.7 × 10^3^	3.6–10.2
Platelets	259 × 10^3^	152–348 × 10^3^
GPT	40 U/L	0–41
GOT	46 U/L	0–40
Creatinine	1.0 mg/dL	0.7–1.2
PT	14.1 s	11.1–14.3
PTT	33.1 s	24–36
INR	1.18	0.8–1.2

Electrocardiography revealed a normal sinus rhythm. Transthoracic echocardiography revealed borderline left ventricular hypertrophy, without significant structural or functional cardiac abnormalities.

Fine‐needle aspiration cytology (FNAC) of the mass suggested a pleomorphic adenoma, with schwannoma considered as the differential diagnosis.

### 2.5. Clinical Decision‐Making

A multidisciplinary discussion involving anesthesia, ENT, and maxillofacial surgery teams concluded that the patient was at high risk for difficult airway management following the induction of general anesthesia because of significant upper airway distortion caused by the tumor.

ATI is therefore considered the safest strategy because it preserves spontaneous ventilation until the airway is secured.

Flexible fiberoptic bronchoscopy and videolaryngoscopy, commonly recommended for ATI, were unavailable at our institution. Alternative techniques, including retrograde intubation and elective tracheostomy under local anesthesia, were considered but reserved as rescue options because they are more invasive and deemed unnecessary if ABNI proved successful.

Following multidisciplinary consultation and informed discussions with the patient, ABNI was selected as the primary airway management technique. An experienced ENT surgeon remained immediately available throughout airway management, and the equipment for emergency surgical tracheostomy was prepared before any airway intervention.

### 2.6. Therapeutic Intervention (Airway Management)

The patient fasted according to the institutional protocol and was transferred to the operating room without premedication to preserve spontaneous ventilation and to facilitate neurological cooperation during awake intubation.

Standard ASA monitoring, including noninvasive blood pressure, three‐lead electrocardiography, pulse oximetry, capnography, and temperature monitoring, was performed before airway manipulation. The patient was preoxygenated with 100% oxygen for 5 minutes using a face mask.

Airway topical anesthesia was performed in a stepwise manner. The patient gargled 5 mL of 2% lidocaine solution (100 mg) for approximately five minutes to anesthetize the oral cavity and oropharynx, while avoiding swallowing the solution. Intravenous lidocaine (80 mg) was administered immediately before intubation to suppress the airway reflexes. Atropine (0.2 mg intravenously) was administered intravenously to reduce airway secretion. The left nostril was prepared using adrenaline‐soaked gauze to achieve mucosal vasoconstriction and to reduce the risk of epistaxis. Water‐soluble lubricant (KY Jelly) was applied to the endotracheal tube before insertion.

A lubricated 6.0‐mm internal diameter cuffed endotracheal tube was gently advanced through the left nostril, while the patient maintained spontaneous breathing. Tube advancement was synchronized with the patient’s inspiratory effort. Correct tracheal placement was identified by maximal breath sounds through the tube, condensation within the tube during respiration, and subsequent confirmation by bilateral chest auscultation and continuous capnography after the induction of general anesthesia.

Tracheal intubation was successfully achieved on the first attempt, without oxygen desaturation, airway trauma, significant epistaxis, or hemodynamic instability.

After confirmation of successful tracheal intubation, general anesthesia was induced using intravenous propofol (100 mg), pethidine (50 mg), tramadol (50 mg), dexamethasone (4 mg), and vecuronium (3 mg). Anesthesia was maintained with isoflurane (0.5–2.0 minimum alveolar concentration) in oxygen and intermittent vecuronium supplementation. Mechanical ventilation was instituted using volume‐controlled ventilation with a tidal volume of 500 mL and a respiratory rate of 12 breaths/min.

Surgical excision was completed uneventfully (Figure 3). The estimated blood loss was approximately 500 mL, which was well below the calculated allowable blood loss. The patient received 1000 mL of isotonic crystalloid solution intraoperatively, and the urine output was approximately 300 mL. The intraoperative hemodynamic parameters were stable (Table 3).

**FIGURE 3:**
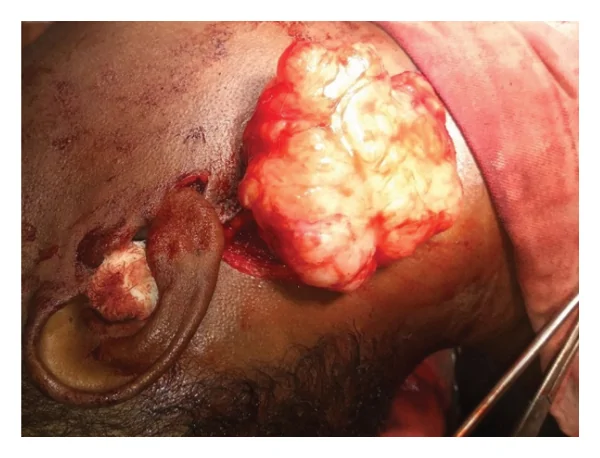
Intraoperative photograph demonstrating complete surgical excision of the giant parapharyngeal space tumor through a transcervical surgical approach. Intraoperative parapharyngeal space tumor surgical excision. Abbreviation: PPS, parapharyngeal space. Written informed consent for the publication of clinical details and accompanying images was obtained from the patient.

**TABLE 3:** Intraoperative hemodynamic parameters.

Parameter	After induction	Intraoperative range	End of procedure
BP	115/80 mmHg	SBP 100–128, DBP 65–80	125/89 mmHg
HR	112 bpm	88–110 bpm	107 bpm
SpO_2_	99%	98%–100%	100%
ETCO_2_	37 mmHg	36–38 mmHg	37 mmHg
Temperature	36°C	35°C–36°C	35°C

The neuromuscular blockade was reversed using neostigmine (2.5 mg) and atropine (1 mg). After complete recovery of spontaneous ventilation and protective airway reflexes, the trachea was extubated uneventfully.

### 2.7. Outcome and Follow‐Up

The patient was transferred to the postanesthesia care unit (PACU), where he remained hemodynamically stable without respiratory compromise or neurological deficits. After 60 min of observation, the patient was transferred to the surgical ward.

The postoperative course was uneventful, and no airway‐related complications, postoperative bleeding, aspiration, or respiratory events were observed. The patient was discharged on postoperative day six in satisfactory clinical condition. The overall patient timeline is presented in detail in Table 4.

**TABLE 4:** Patient timeline.

Time	Clinical event
6 months before admission	Progressive painless intraoral swelling first noticed
Following months	Gradual increase in tumor size with worsening dysphagia, snoring, and oral airway obstruction
3 days before surgery	Hospital admission for preoperative evaluation
Preoperative assessment	Difficult airway anticipated following multidisciplinary evaluation
Day of surgery	Awake blind nasal intubation successfully performed
Intraoperative	Complete surgical excision of giant parapharyngeal space tumor
Postoperative Day 0	Uneventful recovery and transfer to PACU
Postoperative Day 6	Discharged home without complications
Follow‐up	No postoperative airway complications reported

### 2.8. Histopathological Findings

Histopathological examination of the excised specimen demonstrated pleomorphic adenoma/schwannoma, confirming a definitive diagnosis.

## 3. Discussion

Airway management remains the principal anesthetic challenge in patients with giant PPS tumors because progressive distortion of the upper airway anatomy may increase the risk of difficult facemask ventilation, difficult tracheal intubation, and, in severe cases, a CICO scenario following induction of general anesthesia. Consequently, comprehensive preoperative airway assessment and individualized airway planning are essential to minimize perioperative morbidity and mortality [1–6, 11–14].

The uniqueness of this case lies in the successful use of ABNI to secure the airway of a patient with a giant PPS tumor in a resource‐limited hospital, where advanced airway devices, including flexible fiberoptic bronchoscopes and videolaryngoscopes, were unavailable. Although ABNI has largely been replaced by fiberoptic‐ and video‐assisted ATI in contemporary anesthetic practice, this case demonstrates that the technique may still have a role in carefully selected patients when performed by experienced clinicians with meticulous preparation and an immediately available rescue airway plan [5, 6, 14–18].

The current ASA and DAS guidelines recommend ATI whenever difficult airway management is anticipated, and loss of spontaneous ventilation could result in catastrophic airway obstruction. Importantly, these guidelines emphasize the principle of awake airway management rather than the recommendation of a single intubation device. Flexible fiberoptic bronchoscopy remains one of the most widely used techniques because it allows the continuous visualization of airway structures while maintaining spontaneous ventilation. However, videolaryngoscopy has emerged as an equally acceptable option for appropriately selected patients, depending on patient anatomy, equipment availability, and operator expertise [5–9, 12, 15].

In our institution, neither flexible fiberoptic bronchoscopy nor videolaryngoscopy is available because of resource limitations. Therefore, maintaining spontaneous ventilation during airway management became the primary objective when selecting an airway technique that could be safely performed using the available equipment and local expertise. ABNI was selected after a multidisciplinary discussion because the patient remained cooperative, the nasal passage was considered suitable for tube insertion, and emergency surgical tracheostomy could be performed immediately if airway management failed.

Several alternative airway management strategies were considered before selecting the ABNI. Awake fiberoptic intubation was preferred according to international guidelines but was not feasible because the equipment was unavailable. Similarly, awake videolaryngoscopy was impossible for the same reason. Elective tracheostomy under local anesthesia would have provided a definitive airway but was considered unnecessarily invasive as the primary strategy. Retrograde wire‐guided intubation was also considered; however, limited institutional experience and availability of a clinician experienced in blind nasal intubation favored ABNI as the initial approach. Importantly, all rescue airway equipment, including a surgical tracheostomy set, were prepared before attempting intubation [5, 6, 12, 14, 16].

Successful awake intubation depends not only on technical expertise but also on adequate airway preparation. In the present case, topical anesthesia was induced using lidocaine gargle combined with intravenous lidocaine, whereas adrenaline‐soaked nasal packing reduced mucosal congestion and minimized the risk of epistaxis during nasal tube passage. Atropine was administered to reduce airway secretions and improve the procedural conditions. Maintaining spontaneous ventilation through airway instrumentation provides an additional safety margin by preserving oxygenation, which can be prolonged or initially unsuccessful [5, 6, 8, 9, 12].

The selection of anesthetic drugs also reflects the airway strategy. General anesthesia was intentionally deferred until the correct endotracheal tube placement was confirmed. Propofol was subsequently administered for induction because of its rapid onset and favorable recovery profile. Vecuronium was chosen to facilitate controlled mechanical ventilation during surgery, owing to its intermediate duration of action and cardiovascular stability. Dexamethasone was administered to reduce airway edema, postoperative nausea, and vomiting, whereas pethidine and tramadol provided multimodal perioperative analgesia. Delaying the administration of hypnotic agents and neuromuscular blockers until the airway has been secured minimizes the risk of complete airway obstruction during induction [5, 6, 8, 12].

Although ABNI can be lifesaving under carefully selected circumstances, its limitations should be acknowledged. Compared with fiberoptic‐guided techniques, blind nasal intubation is associated with lower overall success rates and a higher incidence of complications, including epistaxis, mucosal trauma, turbinate injury, repeated intubation attempts, and inadvertent esophageal intubation. Published studies have reported variable success rates depending on operator experience, patient selection, and the airway anatomy. Consequently, ABNI should not be considered as a routine first‐line technique in which modern airway devices are available. Instead, its role may be limited to carefully selected patients in resource‐constrained settings, where clinicians possess appropriate expertise and comprehensive rescue airway plans are in place [5–9, 11, 12].

Several factors likely contributed to the favorable outcomes in this patient. First, a thorough preoperative airway assessment identified the potential for airway difficulty before anesthesia induction. Second, spontaneous ventilation was maintained throughout the airway instrumentation. Third, multidisciplinary planning involving anesthesiologists and ENT surgeons ensured the immediate availability of emergency surgical airway interventions. Fourth, meticulous airway topicalization and nasal preparation facilitate atraumatic tube passage. Finally, intubation was performed by an experienced anesthetist familiar with the technique, reducing the likelihood of repeated attempts and airway trauma [5, 6, 12–14].

A final histopathological diagnosis should also be considered when interpreting this case. Preoperative FNAC suggested a pleomorphic adenoma with schwannoma as the differential diagnosis. The final postoperative histopathological diagnosis should be reported because it confirms the definitive diagnosis and provides important information regarding the tumor origin, biological behavior, and prognosis. This information is particularly relevant for head and neck surgeons and pathologists and should be included if available [1–4, 17–23].

This study has several limitations. As a single case report, the comparative safety or effectiveness of ABNI relative to other awake intubation techniques cannot be established. In addition, advanced imaging findings, formal difficult airway risk scores, and long‐term postoperative follow‐up were unavailable, limiting the comprehensive assessment of airway anatomy and long‐term clinical outcomes. Therefore, the findings should be interpreted cautiously and should not be generalized to all patients with PPS tumors or anticipated difficult airways [10, 24].

Nevertheless, this report provides practical evidence that may be valuable for anesthesiologists practicing in low‐resource environments, where advanced airway equipment is unavailable. This highlights the importance of careful patient selection, comprehensive airway assessment, maintenance of spontaneous ventilation, multidisciplinary planning, and the immediate availability of rescue surgical airway techniques. Future studies involving larger case series or multicenter experience are required to better define the role of ABNI within contemporary difficult airway management algorithms [5, 6, 11–14, 25].

Although this report describes only a single patient, it demonstrates that ABNI may remain a feasible airway management option for selected patients with anticipated difficult airways in resource‐limited settings, where fiberoptic bronchoscopes and videolaryngoscopes are unavailable. Successful outcome in this case depended on appropriate patient selection, operator experience, meticulous airway preparation, maintenance of spontaneous ventilation, and readiness to perform emergency surgical airway access. These principles, rather than the specific intubation technique itself, are broadly applicable to difficult airway management across diverse healthcare settings [5, 6, 11–14, 25].

### 3.1. Limitations

This report describes the management of a single patient and, therefore, cannot establish the comparative safety or effectiveness of ABNI. In addition, advanced imaging findings, formal difficult airway risk scores, and long‐term follow‐up data were not available. Consequently, the findings should be interpreted cautiously and should not be generalized to all patients with PPS tumors.

## 4. Conclusion

This case demonstrates that ABNI can be a feasible airway management option in carefully selected patients with giant PPS tumors when advanced airway devices such as flexible fiberoptic bronchoscopes and videolaryngoscopes are unavailable. Successful airway management in this patient was facilitated by a comprehensive preoperative airway assessment, maintenance of spontaneous ventilation during intubation, meticulous airway topicalization, multidisciplinary planning, and immediate preparedness for emergency surgical airway access.

Although ATI remains the recommended approach for anticipated difficult airways, the choice of technique should be individualized according to the patient anatomy, available equipment, and clinician expertise. This case illustrates that ABNI may serve as a practical alternative in selected resource‐limited settings when performed by experienced practitioners using an established rescue airway strategy.

In a single case report, these findings should not be interpreted as evidence of the superiority or routine use of ABNI. Further prospective studies and larger case series are needed to better define its role in contemporary difficult airway management algorithms, particularly in low‐resource healthcare environments.

### 4.1. Generalizability Statement

This report describes the successful management of a single patient and therefore has inherent limitations regarding external validity. Nevertheless, the principles illustrated in this case, including comprehensive airway assessment, preservation of spontaneous ventilation during awake airway management, multidisciplinary collaboration, and preparation for emergency surgical airway access, are broadly applicable to the management of anticipated difficult airway. In healthcare settings where advanced airway technologies are unavailable, carefully selected patients may benefit from alternative awake intubation techniques performed by experienced clinicians within a structured, difficult airway management plan.

### 4.2. Patient Perspective

Following recovery, the patient expressed satisfaction with perioperative care and reported that the procedure and postoperative recovery were well explained. The patient was pleased with a successful surgical outcome and reported an improvement in swallowing and oral airway obstruction after surgery.

### 4.3. Key Learning Points

• Giant PPS tumors may present a significant risk of difficult airway management because of distortion of the upper airway anatomy.
• Comprehensive preoperative airway assessment and multidisciplinary planning are essential for safe perioperative management.
• ATI is recommended for anticipated difficult airways when loss of spontaneous ventilation may be hazardous.
• In resource‐limited settings, where flexible fiberoptic bronchoscopy and videolaryngoscopy are unavailable, ABNI may be considered in carefully selected patients when performed by experienced clinicians.
• Preparation for emergency surgical airway access should always be accompanied by airway management in patients with anticipated difficult intubations.

**Nomenclature**
ABNI: Awake blind nasal intubation
ASA: American Society of Anesthesiologists
ATI: Awake tracheal intubation
BMI: Body mass index
SCARE: Surgical CAse REport guideline
CICO: Cannot intubate, cannot oxygenate
DAS: Difficult Airway Society
ECG: Electrocardiogram
ENT: Ear, nose, and throat
ETCO_2_
: End‐tidal carbon dioxide
ETT: Endotracheal tube
FNAC: Fine‐needle aspiration cytology
INR: International normalized ratio
MAC: Minimum alveolar concentration
NIBP: Noninvasive blood pressure
OR: Operating room
PACU: Postanesthesia care unit
PPS: Parapharyngeal space
PT: Prothrombin time
PTT: Partial thromboplastin time

## Author Contributions

Markos Muka: conceptualization, patient management, data collection, initial manuscript preparation, clinical supervision, critical revision of the manuscript, and interpretation of clinical findings; Aschalew Besha: clinical interpretation and manuscript review; Kanbiro Gedeno: conceptualization, airway management, literature review, manuscript drafting, critical revision, final approval of the manuscript, and corresponding author.

## Funding

The authors received no specific funding for this work.

## Disclosure

All authors have read and approved the final manuscript and agreed to be accountable for all aspects of the work.

## Ethics Statement

According to the institutional policy of Hawassa University Comprehensive Specialized Hospital, individual case reports describing routine clinical care do not require formal institutional review board approval. Written informed consent for publication was obtained from the patient.

## Consent

Written informed consent was obtained from the patient for the publication of this case report and all accompanying clinical images. A copy of the written consent form is available for review by the editor‐in‐chief upon reasonable request.

## Conflicts of Interest

The authors declare no conflicts of interest.

## Supporting Information

Additional supporting information can be found online in the Supporting Information section.

## Supporting information


**Supporting Information** This case report was prepared in accordance with the SCARE (Surgical CAse REport) reporting guidelines [10]. The completed SCARE checklist is provided in Supporting File (SCARE‐Checklist‐2025.docx).

## Data Availability

Data supporting the findings of this case report are included in the published article. Additional anonymized clinical information is available from the corresponding author upon reasonable request, while maintaining patient confidentiality.
